# Identify the therapeutic role and potential mechanism of α-cyperone in diminished ovarian reserve based on network pharmacology, molecular docking, Lip-MS and experimental validation

**DOI:** 10.3389/fphar.2025.1658536

**Published:** 2026-01-15

**Authors:** Jingwen Guo, Xitang Yang, Xue Chen, Rong Hu, Hua Guo

**Affiliations:** 1 Department of Gynecology, General Hospital of Ningxia Medical University, Yinchuan, Ningxia, China; 2 The First Clinical Medical College, Ningxia Medical University, Yinchuan, Ningxia, China; 3 Ningxia Key Laboratory of Clinical and Pathogenic Microbiology, General Hospital of Ningxia Medical University, Yinchuan, Ningxia, China; 4 Reproductive Medicine Center, General Hosptial of Ningxia Medical University, Yinchuan, Ningxia, China; 5 Ningxia Key Laboratory of Stem Cells and Regenerative Medicine, Ningxia Medical University, Yinchuan, Ningxia, China

**Keywords:** α-cyperone, diminished ovarian reserve, network pharmacology, molecular docking, experimental validation, limited proteolysis–mass spectrometry

## Abstract

**Background:**

The presence of diminished ovarian reserve (DOR) poses a significant threat to female fertility, with no current effective treatment available. Inflammation plays pivotal roles in the pathogenesis of DOR. α-Cyperone (AC) exhibits notable anti-inflammatory and anti-oxidative properties; however, its potential for improving DOR remains unexplored.

**Methods:**

The PubChem, PharmMapper, and SwissTargetForecast databases were queried to retrieve biochemical information and drug targets for AC. The identification of disease targets for DOR involved referring to the OMIM and Genecards databases. AC’s therapeutic targets against DOR were determined by examining the overlap between drug targets and disease targets. To analyze GO function enrichment, KEGG pathway, and disease association, the Metascape database was utilized. The results were then visualized using Cytoscape software. Receptor-ligand interaction between AC and target sites was validated through molecular docking investigations utilizing Pymol and AutoDock program software. The effect of AC on granule cell function was verified in CTX-induced DOR granule cell model. The actual AC-binding proteins in the cells were identified by Lip-MS, and the effects of AC on target protein genes were verified by RT-qPCR.

**Results:**

Following the integration of 466 drug targets with 1,529 disease targets, we identified 257 AC targets for the treatment of DOR. We recorded the top 20 enriched biological processes, molecular functions, and KEGG pathways that potentially contribute to the anti-DOR effect of AC. Employing the MCC algorithm, we identified key TOP22 proteins. The docking studies revealed that AC binds strongly to all 22 proteins studied. The CTX-induced DOR granule cell model was successfully established, which was verified by detecting the levels of AMH, ROS, MMP and cell viability, indicating that AC enhanced the function of DOR granule cells. The abnormal expression patterns of MAP2K1, AKT1, ESR2, ERBB2, CDH1, CYP19A1, ESR1 and MAPK8 genes were also reversed. In addition, the binding of AC to MAP2K1, GSK3B and MAPK14 was verified by Lip-MS experiments.

**Conclusion:**

AC can improve CTX-induced KGN proliferation and improved the function of KGN cell. The mechanism may be due to the targeted binding ability of AC to domains of MAP2K1, MAPK14 and GSK3B. AC’s potential therapeutic targets are comprehensively explored in this study, as well as theoretical support for its use in the treatment of DOR is provided.

## 1 Introduction

Diminished ovarian reserve (DOR) is a pathological state characterized by the gradual decline in both quantity and quality of oocytes, making it a common age-associated condition affecting female reproductive health. It accounts for around 20% of all ovarian illness (Jiao et al., 2021; Practice Committee of the American Society for Reproductive, 2012). This condition significantly impacts women’s fertility potential and overall wellbeing, exerting substantial implications on their reproductive health and overall quality of life.

Accumulating evidence has suggested that the pathogenic mechanism of DOR is associated with several elements, including advanced age, enetic predisposition, infections, immune, environmental factors and iatrogenic causes (Li et al., 2023; Greene et al., 2014; He et al., 2023). However, even after excluding these risk factors, the etiology of DOR remains unknown in some patients. Recent studies have demonstrated that chronic aseptic inflammation and oxidative stress play pivotal roles in the development of DOR (Luderer, 2014; Snider and Wood, 2019; de Souza et al., 2015; Senates et al., 2013). The findings revealed significantly elevated levels of inflammatory markers in both serum and follicular fluid among DOR patients compared to healthy women (Yang et al., 2018). The aforementioned suggests that the inflammatory state of the follicular microenvironment may have an impact on oocyte quality. It has been shown that increased inflammation of granulosa cells within the follicle is negatively correlated with oocyte quality. Intra-follicular TNF-α has been found to potentially enhance IL-1 and IL-6 secretion by up-regulating the NF-κβ pathway in granulosa cells, consequently leading to a reduction in oocyte quality (Li et al., 2019). Previous research has suggested that individuals with DOR exhibited notably elevated levels of NLRP3, IL-1β, and caspase-1 expression in both oocytes and granulosa cells. After inhibiting inflammasome activity, there was a rise in anti-Mullerian hormone (AMH) levels and autophagy rates in the ovaries, leading to noticeable improvement in ovarian function (Navarro-Pando et al., 2021). Compared to mice with the wild-type genotype, mice lacking TNF-α demonstrate increased multiplication levels in granulosa cells within the ovaries, a decreased incidence of follicular atresia, and improved fertility. These findings suggest that TNF-α plays a crucial role in facilitating ovarian function by controlling apoptosis in granulosa cells (Cui et al., 2011). In addition, oxidative stress has been found to induce ovarian endocrine dysfunction resulting in compromised oocyte quality along with granulosa cell apoptosis leading to follicular atresia–another significant cause for ovarian function impairment (Luderer, 2014).

Current clinical treatment strategies for DOR encompass hormone replacement therapy, assisted reproductive technology, and lifestyle interventions. However, these approaches are limited in their applicability, cost implications, significant side effects, and potential risks (Jiao and Bukulmez, 2021), thereby posing a substantial clinical challenge in the treatment of DOR (Zhang et al., 2023). Considering the current absence of efficacious clinical remedies, it is imperative to expedite the development of pharmaceutical interventions targeting DOR for both preventive and therapeutic purposes.

As a traditional Chinese medicine, Xiangfu (*Cyperi Rhizoma*) has a long history and is widely used in clinic. Modern research has shown that α-Cyperone, which is the main chemical component of *Cyperi Rhizoma*, has exhibited pharmacological activities including antimicrobial, antioxidant, neuroprotective, anti-hemolytic, and anti-inflammatory effects (Taheri et al., 2021). A research study has shown that α-Cyperone exhibits neuroprotective effects on dopaminergic neurons and reduces neuroinflammation in a rat model of Parkinson’s disease induced by LPS. The suppression of the NF-κβ signaling pathway and activation of the Nrf2/HO-1 signaling pathway are responsible for accomplishing this (Huang et al., 2023). According to the study conducted by Xueshibojie L et al., α-Cyperone effectively alleviates acute lung injury induced by LPS in mice by activating SIRT1 through targeting the NF-κβ and NLRP3 signaling pathways (Liu et al., 2019). Xiao-Dong P. et al. Conducted a study which revealed that α-Cyperone has the ability to regulate the PI3K/Akt/mTOR signaling pathway mediated by ROS, resulting in the suppression of HeLa cell proliferation in cervical cancer (Pei et al., 2020). However, the potential role of α-Cyperone in ovarian protection in DOR has not yet been investigated in either domestic or international studies.

The remarkable advancements in the realm of bioinformatics have given rise to network pharmacology as an influential tool for comprehensively grasping the workings of intricate drug systems. One notable characteristic of traditional Chinese herbal formulations is their ability to simultaneously target numerous pathways, processes, and targets. The core principle of network pharmacology involves utilizing diverse databases to construct interconnected networks that reveal the connections between chemical compounds, drugs, and targets. This method enables the conversion of complex mechanisms related to drugs with multiple components and targets into visually comprehensible graphical representations, simplifying the systematic analysis of interactions between target proteins and the anticipation of molecular mechanisms that underlie Chinese herbal treatments for diseases (Chen et al., 2023; Huang, 2023). Many research studies have utilized network pharmacology techniques to understand how drugs work in treating diseases, while also demonstrating the potential of this methodology for accelerating drug discovery and development (Wu et al., 2022). In addition, chemical proteomics can reveal the direct binding between small molecules and proteins, and is often used to find the action targets of small molecules such as drugs and endogenous metabolites. Traditional chemical proteomics methods have limited the application of probe affinity due to the difficulty in preparation of chemical probes. Limited proteolysis–mass spectrometry (LiP–MS) is a new class of chemical proteomics method: after the drug binds to the target protein, it will block the restriction site of the protein, and then cause the target protein sequence to be “resistant” to enzyme digestion, resulting in changes in the sequence and abundance of the peptide product produced by enzyme digestion. Therefore, sequence identification and quantification of protease cleavage products by mass spectrometry can distinguish which protein sequences are binding targets for small molecules. Therefore, the chemical proteomics method based on limited enzyme digestion does not require chemical probe preparation, which is highly operable and can precisely locate the target sequence. (Piazza et al., 2018).

Considering the role of chronic inflammation and oxidative stress in the pathogenesis of DOR, as well as the widespread utilization of α-Cyperone for its anti-inflammatory and antioxidant properties, we postulate that α-Cyperone may hold significant therapeutic potential in managing reduced ovarian reserve and preserving ovarian function. In this study, we employed network pharmacology and Lip-MS to identify potential targets associated with α-Cyperone’s efficacy against DOR, followed by subsequent validation of its underlying mechanism.

## 2 Materials and methods

### 2.1 Drug targets of AC

The PubChem database is a publicly accessible repository that offers an extensive collection of chemical information at no cost, thereby providing a comprehensive resource for drug discovery endeavors. (Kim, 2016). Hence, the PubChem database was employed to extract pertinent details regarding AC, aiding in the discovery of potential targets. SwissTargetPrediction, a web-based tool that utilizes both 2D and 3D similarity metrics of known ligands, effectively forecasts target molecules with biological activity (Gfeller et al., 2014). PharmMapper proves to be a valuable tool for the discovery of promising drug targets. It is an online tool that utilizes reverse pharmacophore matching and allows for compound querying against an internal database containing a model of pharmacophores (Wang et al., 2017). Potential targets were predicted independently with these two web servers: SwissTargetPrediction (2-D/3-D ligand similarity, probability ≥0.7) and PharmMapper (reverse pharmacophore matching, Z-score ≥0.9). Only proteins identified by both tools were retained, and their gene symbols were standardised with UniProt (UniProt, 2023).

### 2.2 Therapeutic targets for DOR and identification of drug-disease intersection targets

The Genecards database functions as an extensive and reliable collection of annotated data related to genes. (Safran et al., 2010). It excels in its capacity to comprehensively calculate the relevance score of diseases and genes, enabling it to identify gene-associated diseases with precision. Meanwhile, OMIM (Online Mendelian Inheritance in Man) functions as a highly dependable and current research tool, meticulously elucidating the intricate relationships between human genes and phenotypes (Amberger et al., 2015). Therefore, DOR-associated genes were extracted from OMIM (curated entries) and GeneCards (relevance score ≥10) by searching the term “diminished ovarian reserve”. The final AC-versus-DOR target list was generated as the intersection of the two drug-target and two disease-target sets with Venny 2.1, ensuring that only well-supported candidates were analysed further.

### 2.3 Establishing a network for the protein-protein interaction (PPI)

To explore the association between overlapping genes, we utilized the STRING database and specified “*Homo sapiens*” as the species to import the predicted therapeutic targets (Szklarczyk et al., 2021). A confidence score threshold of ≥0.7 was applied to obtain a Protein-Protein Interaction (PPI) network diagram, which was then exported in TSV format and subsequently refined using Cytoscape 3.10.1 for further analysis (Liang et al., 2024). The Cytoscape software, an application for visualizing and analyzing network biology (Huang, 2023), was utilized to calculate node parameters and visualize molecular connections in the network diagram. To isolate significant targets prior hub gene identification, calculate the significant values by using the cytoNCA plugin to assess degree centrality, between centrality and closeness centrality. To investigate the associations among target genes, we utilized the cytoHubba plugin to assess node degree centrality. Afterwards, we chose the top 20 nodes and arranged them in the inner circle for visualization purposes. Lastly, we employed the MCC algorithm to identify and screen the top 20 genes (Chin et al., 2014).

### 2.4 GO and KEGG enrichment

The therapeutic targets identified for treatment of DOR in AC were examined using Kyoto Encyclopedia of Genes and Genomes (KEGG) pathway analysis and Gene Ontology (GO) enrichment analysis. Biological processes (BP), cellular components (CC), and molecular functions (MF) terms were retrieved with the Metascape database (adjusted P < 0.01) (Zhou et al., 2019). To ensure reliability, each target list was simultaneously uploaded to DAVID 6.8 and STRING (Szklarczyk et al., 2021; Huang da et al., 2009), only terms recovered by at least two independent platforms and exhibiting identical leading genes were retained. Enrichment results were visualised with bubble plots generated through the bioinformatics web server (https://www.bioinformatics.com.cn) (Liang et al., 2024; Gene Ontology, 2015).

### 2.5 Obtained the key 22 targets of α-cyperone for DOR

The STRING database was utilized to import all targets that were enriched in the three pathways related to ‘production of female gametes, response to hormones, and sex differentiation’. Subsequently, these targets were imported into Cytoscape 3.10.1 software for network analysis using the MCC algorithm provided by the cytoHubba module. The top 20 key genes were selected again. After merging and eliminating duplicate entries from the two sets of 20 primary objectives acquired, a final collection of 22 crucial targets was obtained for subsequent examination.

### 2.6 Molecular docking validation of the binding capacity between AC and targets

The 3-dimensional configuration of the AC molecule was acquired from the PubChem database (Kim et al., 2016) and transformed into Mol2 format utilizing Open Babel program (O'Boyle et al., 2011). The template structures of the target proteins were acquired from the UniProt database (UniProt, 2023) and RCSB Protein Data Bank (Chen et al., 2023; Berman et al., 2000). To prepare the target proteins for further analysis, water molecules and original ligands were removed using Pymol before importing them into AutoDock Tools 1.5.7. Subsequently, hydrogenation, charge calculation, and non-polar hydrogen combination were performed. After determining the Grid Box size and genetic algorithm parameters, molecular docking was performed using AutoDock Vina through CMD command characters. Finally, Pymol was utilized to visualize the results (Huang, 2023).

### 2.7 Cell culture and treatment

The KGN cells used in this study were obtained from Procell Life Science Technology Co., Ltd. for *in vitro* research purposes. The KGN cells were cultured in a culture medium composed of DMEM/F12, which was supplemented with 10% FBS (fetal bovine serum). The culture conditions were maintained at a temperature of 37 °C in an environment containing 5% CO_2_, and the culture medium was renewed every 2 days. Upon reaching approximately 80% confluency and displaying favorable condition, the cells were divided into three groups (n = 6 wells per group, three independent passages on different days): The normal control (NC group): complete medium only; CTX model (CTX group): 10 μg/mL cyclophosphamide (Sigma, C3250000) dissolved in serum-free DMEM; α-Cyperone (AC group): pre-treated with varying concentrations α-Cyperone (MedChemExpress, HY-N0710) for 6 h, followed by 10 μg/mL CTX without wash-out. Cyclophosphamide (CTX; C3250000) was purchased from Sigma (United States) and dissolved in DMEM as per manufacturer’s instructions. α-Cyperone was purchased from MedChemExpress (HY-N0710, Monmouth Junction, NJ, United States) and dissolved in Methyl sulfoxide (DMSO; Cat# D8371, Solarbio Life Sciences, Beijing, China), and in the final cultivation system, the content of DMSO should not exceed 0.01%.

### 2.8 Cell viability assay (CCK-8)

The KGN cells were cultured in a 96-well plate with a cell concentration of 1 × 10^4^ cells/mL. Each well was supplemented with 100 µL of cell suspension. A minimum of three replicate wells were included in each experimental group. The plate was then placed in an incubator set at 37 °C and enriched with 5% CO_2_ until the cells reached approximately 80% confluence. Subsequently, the culture medium of the AC groups was aspirated and gradient concentrations of AC were introduced into the wells, followed by an incubation period of 6 hours. CTX (300 μM) was subsequently added to each well in both the CTX and AC groups. The cells were incubated for an extra 24 h in the culture medium. Next, 10 µL of CCK-8 enhancement solution (Meilunbio, China) was added to each well and allowed to incubate for 1 hour before measuring optical density (OD) at 450 nm using a microplate reader according to standard protocols for colorimetric assays.

### 2.9 Elisa

Using the AMH assay kit (Nanjing BYabscience technology Co.,Ltd., BY-EH111084), the optical density (OD) at 450 nm was read and the concentration of AMH in the supernatant of KGN cells was determined by comparing with the standard curve.

### 2.10 ROS levels assay

Intracellular reactive oxygen species (ROS) were quantified using the 2,7-dichlorodihydrofluorescein diacetate (DCFDA) fluorescent probe (Beyotime Biotech, Nanjing, China). A cell suspension containing 2 × 10^5^ cells was exposed to a 50 mM DCFDA solution in pre-warmed PBS for 30 min at 37 °C, following which the fluorescent signal was captured under excitation at 488 nm and emission at 525 nm.

### 2.11 Mitochondrial membrane potential detection

The JC-1 probe was employed to detect mitochondrial depolarization of KGN cells, following the manufacturer’s protocol. Briefly, after the indicated treatments, the cells cultured in 6-well plates were incubated with 5 μg/mL JC-1 for 20 min at 37 °C. The staining solution was removed and cells were washed with JC-1 staining buffer twice. The distribution and fluorescence intensity of JC-1 monomers and aggregates were visualized by fluorescence microscopy, and the images were assessed using the ImageJ software. Mitochondrial depolarization was evaluated by the decrease in the red/green fluorescence intensity ratio.

### 2.12 Reverse transcription-quantitative polymerase chain reaction (RT-qPCR)

The KGN cells in the NC, CTX, and AC groups were subjected to total RNA extraction using an RNA extraction kit (#G3640-50T, Servicebio). The spectrophotometric analysis was conducted to evaluate the concentration and purity of the extracted RNA, measuring absorbance at 260 nm and 280 nm. In order to ensure optimal quality, RNA samples with a D(λ)260/D(λ)280 ratio falling within the range of 1.8–2.0 were stored at −80 °C for future utilization. Subsequently, cDNA synthesis was performed using FastKing gDNA Dispelling RT SuperMix (Tiangen Biotech; Beijing; China), followed by PCR amplification on a lightcycler480II Real-time PCR instrument with SYBR Green PCR master mix (#G3326-05, Servicebio). Meanwhile, the β-actin gene was concurrently assessed as an internal reference in conjunction with the samples. The 2^−ΔΔCT^ method was employed to determine the relative levels of gene expression. All experiments were performed in triplicate parallel assays. The primers utilized in this investigation were custom-designed and synthesized by TsingKe (Wuhan, China), and their specific sequences can be found in Table 1.

**TABLE 1:** Sequences of PCR primers.

Gene symbol	Forward primer (5′–3′)	Reverse primer (5′–3′)
β-actin	TAT​GCT​CTC​CCT​CAC​GCC​ATC​C	GTC​ACG​CAC​GAT​TTC​CCT​CTC​AG
MAP2K1	CAA​TGG​CGG​TGT​GGT​GTT​C	GAT​TGC​GGG​TTT​GAT​CTC​C
GSK3B	AGA​CGC​TCC​CTG​TGA​TTT​ATG​T	CCG​ATG​GCA​GAT​TCC​AAA​GG
MAPK14	GCT​CTC​CAG​ACC​ATT​TCA​GT	AAT​TCC​TCC​AGA​GAC​CTT​GC
SRC	ACT​ACT​CCA​AAC​ACG​CCG​AT	CCC​ATC​CAC​ACC​TCG​CCA​AA
AKT1	CCT​TCA​TCA​TCC​GCT​GCC​TG	TCC​ATC​TCC​TCC​TCC​TCC​TG
ESR2	CTC​CTT​TAG​TGG​TCC​ATC​GC	CCC​TCT​TTG​AAC​CTG​GAC​CA
ERBB2	GCG​AGA​GGT​GAG​GGC​AGT​TA	TCC​CCA​TCA​AAG​CTC​TCC​GGC
CDH1	GAT​AGA​GAA​CGC​ATT​GCC​AC	GGG​TGA​ATT​CGG​GCT​TGT​TG
CYP19A1	GCT​GGA​CAC​CTC​TAA​CAC​GC	AGG​AGA​GCT​TGC​CAT​GCA​TC
ESR1	GGG​AAG​TAT​GGC​TAT​GGA​ATC​TG	TGG​CTG​GAC​ACA​TAT​AGT​CGT​T
MAPK8	TCT​GGT​ATG​ATC​CTT​CTG​AAG​CA	TCC​TCC​AAG​TCC​ATA​ACT​TCC​TT
PARP1	CGG​AGT​CTT​CGG​ATA​AGC​TCT	TTT​CCA​TCA​AAC​ATG​GGC​GAC

### 2.13 Limited proteolysis–mass spectrometry (LiP–MS)

This part of the experiment and data analysis were performed by APT Biotechnology (Shanghai, China).

#### 2.13.1 Protein extraction and enzymatic digestion of peptides

Samples of granulosa cells in the CTX group were collected and homogenized with 1x PBS for protein extraction, and BCA was used for protein quantification. The samples were equally divided, the AC group was treated with 40μM AC, and the CTX group was added with 1.4 mM DMSO, to ensure that the DMSO content in both groups is the same, and the final concentration of DMSO in the system should not exceed 0.01%. (Figure 7A). The treated samples were pretreated with 50 μg/mL proteinase K (PK enzyme) for 15 min and 15ug of protein was subjected to SDS-PAGE. The PK-treated samples were added with denaturing agent (UA/DOC), DTT was added to the final concentration of 20mM, the reaction was performed at 30° for 2 h, then cooled to room temperature, IAA was added to the final concentration of 25mM, shaking at 600rpm for 1min, and dark room temperature for 30min. The UA/DOC concentration was diluted to below 1.5M by adding an appropriate amount of NH4HCO3 buffer (50 mM). Then, 2 μg Trypsin was added to the sample and incubated at 37° C for 16 h. They were desalted and lyophilized and then redissolved in 0.1%FA. Peptide concentration was determined by OD280. Then 2 μg of each peptide was removed and incorporated with appropriate amount of iRT standard peptide for DIA mass spectrometry detection.

#### 2.13.2 Mass spectrometry

DIA analysis was performed using a nanoliter flow rate Vanquish Neo system (Thermo Fisher) for chromatographic separation, and samples separated by nanoliter HPLC were subjected to DIA (data independent) mass spectrometry on an Astral high-resolution mass spectrometer (Thermo Scientific). Detection mode: positive ion, parent ion scanning range was 380–980m/z, primary mass spectrometry resolution was 240,000 at 200 m/z, Normalized AGC Target was 500%, and Maximum IT was 5 m. MS2 used DIA data acquisition mode, set 299 scanning Windows, Isolation Window was 2m/z, HCD Collision Energy was 25ev, Normalized AGC Target was 500%. The Maximum IT is 3 m.

#### 2.13.3 Data analysis

DIA data were processed using Spectronaut software. Software parameters are set as follows: retention time prediction type is set to dynamic iRT, interference on MS2 level correction is enabled, cross run normalization was enabled, and all results had to pass the Q Value cutoff of 0.01 (FDR<1%).

#### 2.13.4 Bioinformatics analysis

The quantitative information of the target protein set was first normalized. Then, the Complexheatmap R package (R Version 3.6.3) was used to simultaneously classify the expression of samples and proteins in two dimensions (distance algorithm: Euclidean, connection mode: Average linkage) and generate hierarchical clustering heatmaps. The target protein set was normalized, and the fuzzy c-means (FCM) algorithm of Mfuzz software was used for fuzzy C-means clustering algorithm analysis. According to the expression trend, the protein set was divided into different expression modules to generate different clusters. Protein domain analysis was performed using the Pfam database, and functional characterization of sequences was performed by running scanning algorithms from the InterPro database in an integrated manner using the InterProScan software package, thereby obtaining domain annotation information of target protein sequences in the Pfam database. GO annotation of the set of target proteins was performed using Blast2GO. KAAS (KEGG Automatic Annotation Server) software was used to perform KEGG pathway annotation of the target protein set. Fisher’s Exact Test was used to compare each GO category (or KEGG pathway, or GO annotation or (or KEGG pathway, or Domain) annotation enrichment analysis was performed on the target protein set.

### 2.14 Statistical analysis

Data are expressed as mean ± SEM of n = 18 replicates per condition (six wells per group in each of three independent experiments). Normality was confirmed for every data set by Shapiro–Wilk test and homoscedasticity by Brown–Forsythe (≥3 groups) or F-test (2 groups); all P > 0.05. Consequently, group means were compared with two-tailed unpaired t-test (two groups) or one-way ANOVA followed by Tukey’s HSD (≥3 groups) in GraphPad Prism nine and R 4.2.0. If either assumption was violated (P ≤ 0.05), Welch’s correction or Kruskal–Wallis test was substituted. Statistical significance was accepted at α = 0.05 and denoted *p < 0.05, **p < 0.01, ***p < 0.001.

## 3 Results

### 3.1 Identification of AC drug and DOR targets

**FIGURE 1:**
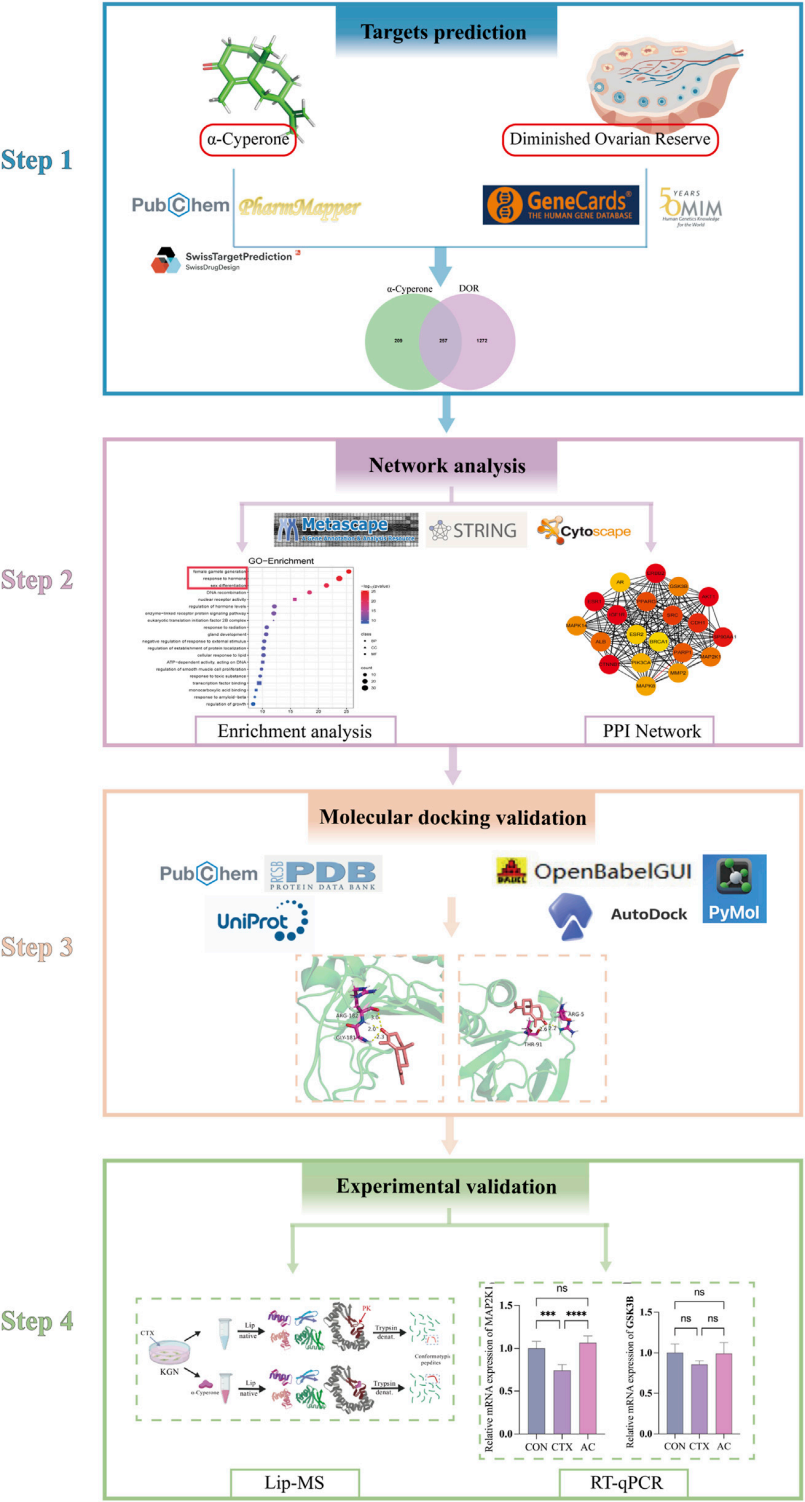
Flowchart of a network pharmacology-based approach to investigate the pharmacologic targets of α-Cyperone in the treatment of DOR.

**FIGURE 2:**
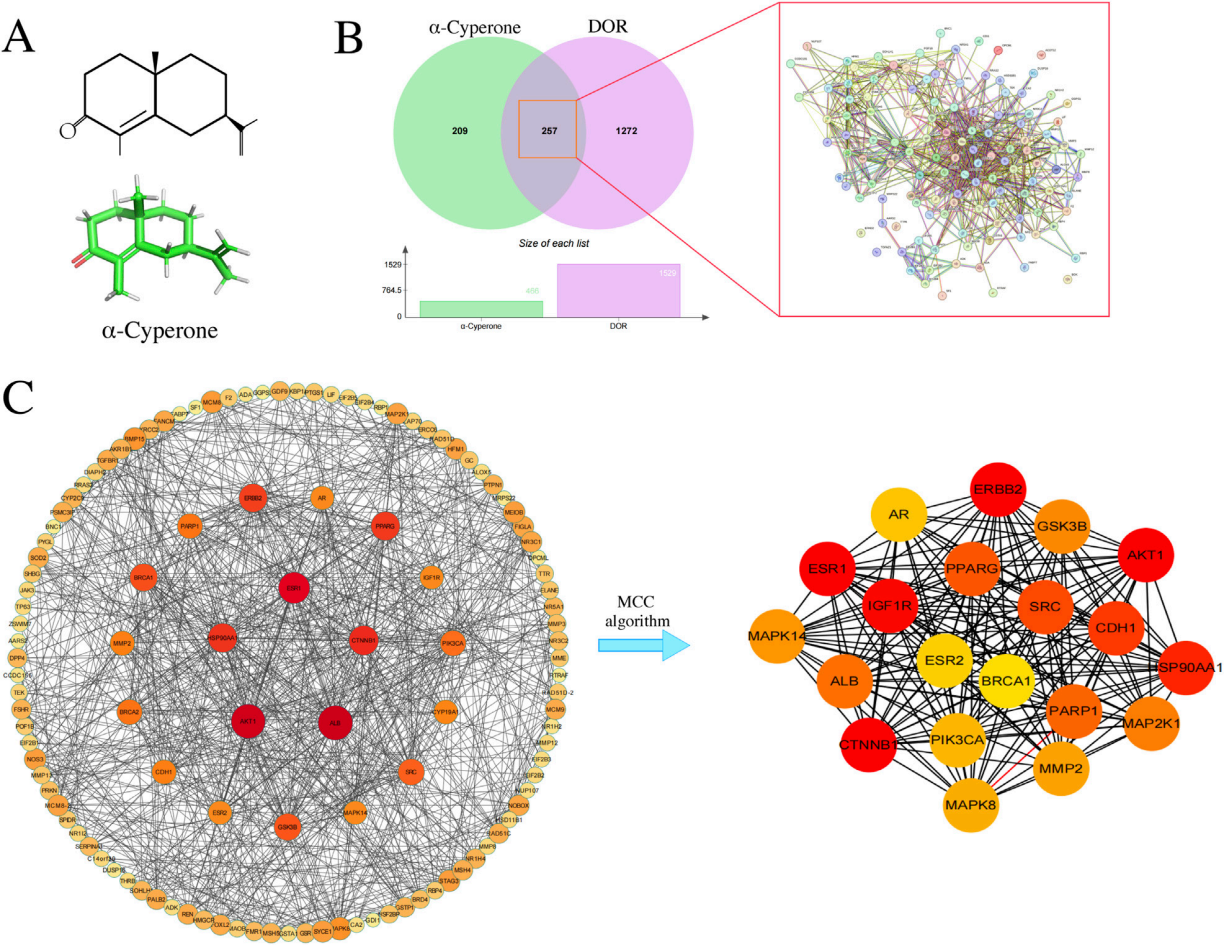
Screening and analysis of potential targets of α-Cyperone on DOR. **(A)** 2D and 3D structure of α-Cyperone. **(B)** Venn diagram of the interaction between α-Cyperone-target and DOR-target. **(C)** PPI Network diagram of target genes. “α-Cyperone target - DOR targe.

The workflow for screening and identifying key targets for AC treatment of DOR is shown in Figure 1. The 2D and 3D configurations, along with associated details of AC, as depicted in Figure 2A, were acquired from the PubChem database (PubChem CID:89528182). Pymol was utilized to generate visual representations of the 3D structure. In order to investigate the potential mechanisms underlying the improvement of DOR by AC, we conducted a search for relevant targets of both AC and DOR, followed by standardizing the obtained gene names using information from the UniProt database. In SwissTargetPrediction and Pharmmapper databases, a total of 198 and 267 AC drug targets were identified respectively. After eliminating duplicates, we selected 466 unique drug targets for further analysis. In Genecards and OMIM databases, there were 1,356 and 202 targets associated with DOR respectively. Following the removal of redundant targets, we obtained a total of 1529 DOR-related targets. Given the scarcity of databases dedicated to identifying targets for both AC and DOR, we constructed separate interaction networks linking AC- and DOR-related targets and assigned distinct color codes to the nodes (targets) retrieved from specific websites (Supplementary Figure S2).

### 3.2 Screening and analysis of key targets of AC on DOR

We analyzed the drug targets for AC and compared them with DOR targets to identify potential therapeutic targets for treating DOR with AC. Our analysis resulted in the identification of a total of 257 potential targets (Figure 2B). The protein interaction network of the remaining genes was established by excluding unrelated genes from the STRING database and enhanced using Cytoscape software. The left side of Figure 2C illustrates the construction of the “AC target-DOR target” network. Subsequently, we applied the MCC algorithm to identify the top 20 genes, as depicted on the right side of Figure 2C, which included AKT1, ESR1, CTNNB1, IGF1R, ERBB2, HSP90AA1, among others. The detailed data regarding the top 20 genes can be found in Table 2.

**TABLE 2:** Information about 22 key target proteins and their molecular docking results with AC.

Targets	Name	Uniprot ID	PDB ID	Classification	Energy (Kcal/mol)	Ligand efficiency
MAP2K1	Dual specificity mitogen-activated protein kinase kinase 1	Q02750	9AY7	Transferase	−6.78	−0.42
GSK3B	Glycogen synthase kinase-3 beta	P49841	4AFJ	Transferase	−6.35	−0.40
MAPK14	Mitogen-activated protein kinase 14	Q16539	1WBV	Transferase	−6.34	−0.40
SRC	Proto-oncogene tyrosine-protein kinase Src	P12931	2H8H	Transferase	−6.34	−0.40
AKT1	RAC-alpha serine/threonine-protein kinase	P31749	1UNQ	Transferase	−6.25	−0.39
ESR2	Estrogen receptor beta	Q92731	3OLL	Hormone receptor	−6.11	−0.38
ERBB2	Receptor tyrosine-protein kinase erbB-2	P04626	3H3B	Immune system	−5.81	−0.36
CDH1	Cadherin-1	P12830	3FF7	Cell adhesion	−5.79	−0.36
CYP19A1	Cytochrome P450 19A1	P11511	5JKV	Oxidoreductase	−5.65	−0.35
ESR1	Estrogen receptor	P03372	1A52	Hormone receptor	−5.51	−0.34
MAPK8	Mitogen-activated protein kinase 8	P45983	4YR8	Transferase	−5.5	−0.34
PARP1	Poly [ADP-ribose] polymerase 1	P09874	6BHV	Transferase	−5.48	−0.34
IGF1R	Insulin-like growth factor 1 receptor	P08069	1IGR	Hormone receptor	−5.45	−0.34
MMP2	72 kDa type IV collagenase	P08253	1CK7	Hyderolase	−5.42	−0.34
PPARG	Peroxisome proliferator-activated receptor gamma	P37231	1FM6	Transcription	−5.32	−0.33
HSP90AA1	Heat shock protein HSP 90-alpha	P07900	3K99	Chaperone	−5.26	−0.33
PIK3CA	Phosphatidylinositol 4,5-bisphosphate 3-kinase catalytic subunit alpha isoform	P42336	2ENQ	Transferase	−5.20	−0.33
BRCA2	Breast cancer type 2 susceptibility protein	P51587	8BR9	Antitumor protein	−5.14	−0.32
ALB	Albumin	P02768	7OV1	Transport protein	−4.89	−0.31
AR	Androgen receptor	P10275	5JJM	Transcription	−4.80	−0.30
CTNNB1	Catenin beta-1	P35222	1G3J	Transcription	−4.78	−0.30
BRCA1	Breast cancer type 1 susceptibility protein	P38398	3COJ	Antitumor protein	−4.75	−0.3

**FIGURE 3:**
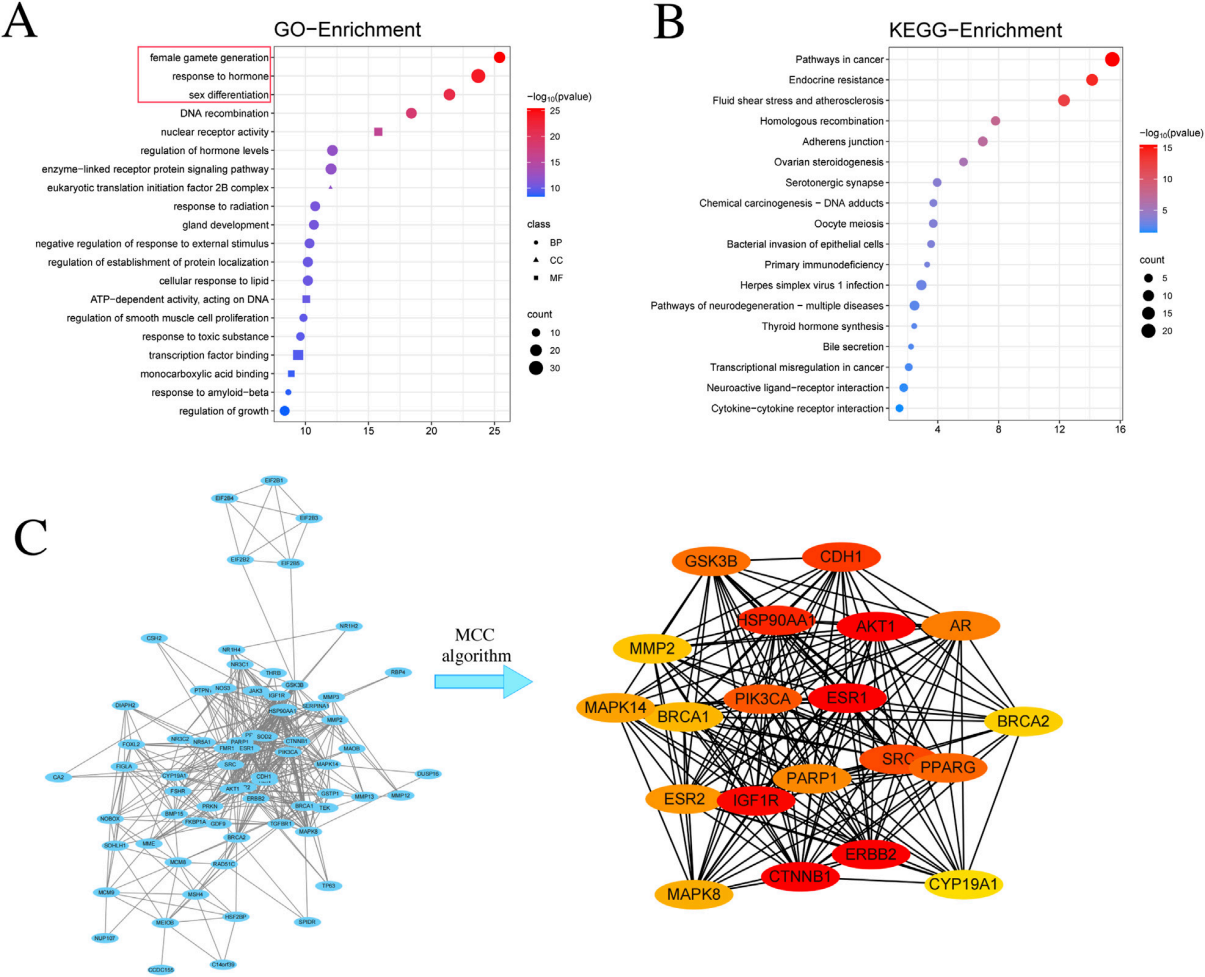
Network Pharmacology Predicts the Potential Mechanisms of α-Cyperone in Treating DOR. **(A)** GO enrichment analysis, including Biological Processes (BP), Cellular Components (CC), and Molecular Functions (MF). **(B)** KEGG enrichment analysis. Bubble size represents the number of enriched genes, and bubble color indicates the P-value. **(C)** Network maps of all crossed target genes in the top three pathways of the GO enrichment analysis were screened, and the top 20 key genes obtained by MCC algorithm.

The Metascape database was utilized to conduct a comprehensive analysis on the targets, revealing significant enrichment in GO terms and KEGG pathways (Figures 3A,B). The findings indicate that AC is essential in multiple biological functions such as the production of female reproductive cells, hormonal responses, sexual differentiation, regulation of hormone levels, and development of glands. Molecular functions include nuclear receptor activity, ATP-dependent activity, acting on DNA, transcription factor binding and monocarboxylic acid binding.

Since DOR patients usually have abnormal sex hormone levels, and the results of GO enrichment suggest that the targets of AC action on DOR are closely related to hormone regulation, we focused on the targets enriched in the biological pathway of “female gamete production, responses to hormone, sex differentiation”. We uploaded all targets enriched in the aforementioned three pathways to the STRING database, followed by their importation into Cytoscape 3.10.1 software, and performed a network analysis using the MCC algorithm of the cytoHubba module, again selecting the top 20 key genes (Figure 3C). After consolidating and eliminating duplicate entries from the initial pool of 20 crucial targets identified in two separate ways, a refined set of 22 key targets was derived for subsequent investigation. Table 2 contains comprehensive data on the 22 primary objectives mentioned.

### 3.3 Validation of binding capacity between AC and 22 therapeutic targets by molecular docking

**FIGURE 4:**
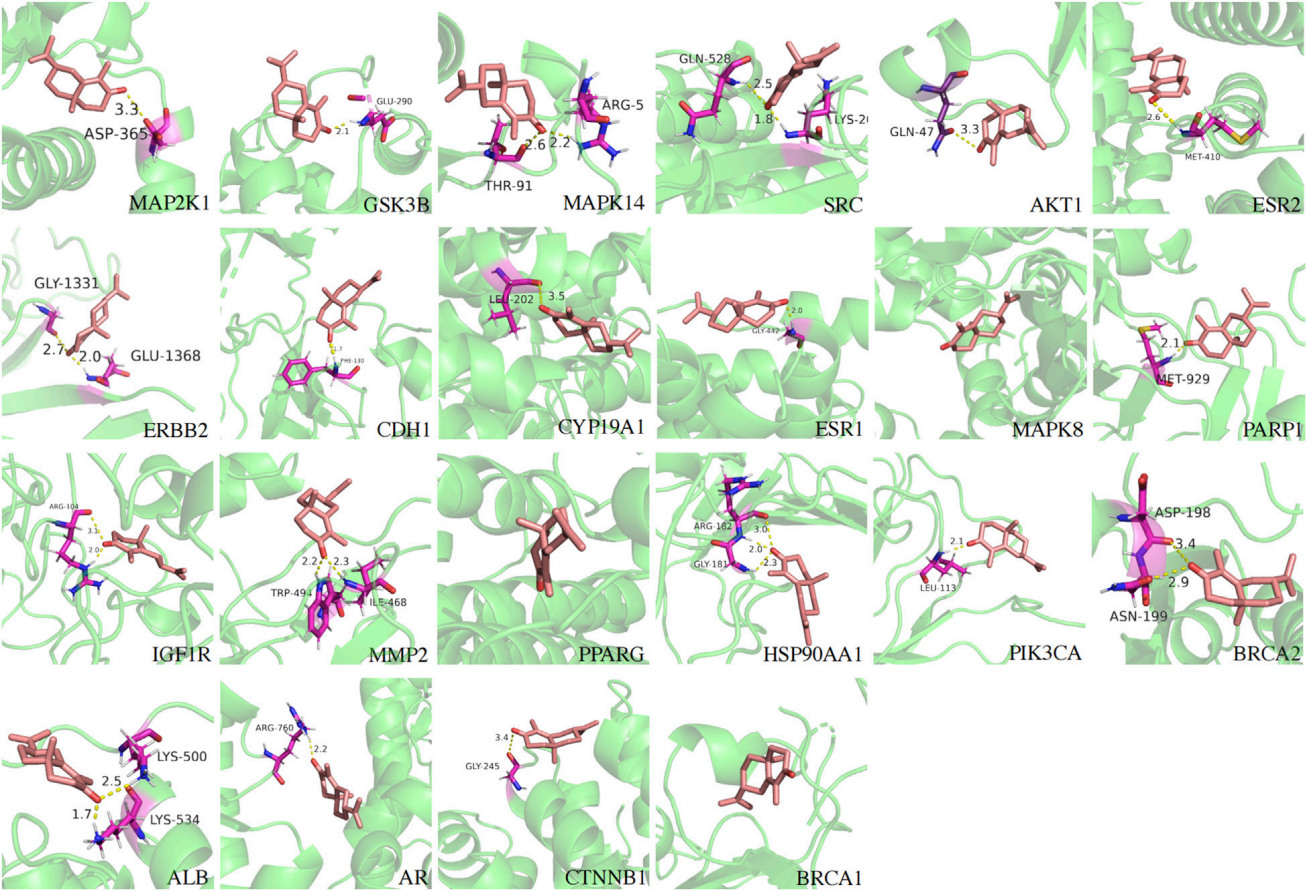
Molecular docking validation of the binding capacity between AC and targets, and shown the 3-dimensional map of the binding sites. AC is shown in violet. Target proteins are displayed as green. The places where AC and the target proteins are connected represent specific docking sites between AC and target proteins and are shown in magenta. AC:α-Cyperone.

The molecular docking analysis was conducted to investigate the interactions between AC and a set of 22 specific genes. Employing AutoDock software, all the docking results were generated and displayed low binding energies (Delta G), indicating strong affinities between the compound and the targets (Figure 4). The molecular docking results of AC with target proteins are shown in Table 2. Notably, all target proteins exhibited robust binding to AC with respective binding energy all <0 kcal/mol, which indicated that AC and each target can bind spontaneously, signifying their significant roles in the molecular mechanism of AC treatment on DOR. The visualizations of the lowest binding energies between each target and AC were created using Pymol. Furthermore, we also employed visualizations to depict the surface configurations of the target proteins in the molecular docking outcomes. Notably, it is evident that all AC molecules are situated within the active pockets of their respective proteins (Supplementary Figure S1).

### 3.4 Effect of AC on CTX-induced KGN injury model

**FIGURE 5:**
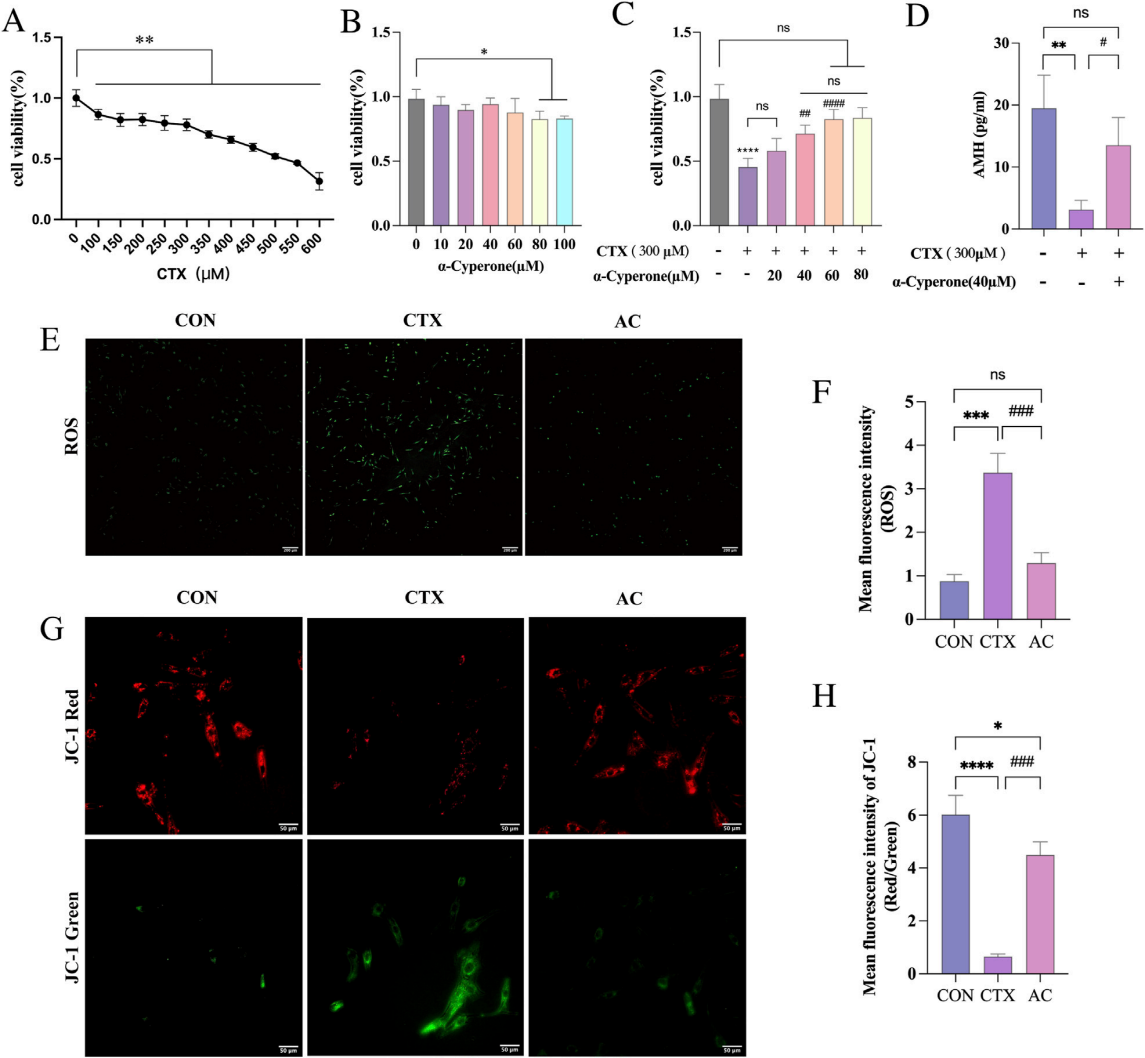
Effect of AC on CTX-induced KGN injury model. **(A)** KGN cell viability was assayed by CCK-8 after treating KGN cells with graded concentrations of CTX alone for 24 h. **(B)** KGN cell viability was assayed by CCK-8 after treating KGN cells with graded concentrations of AC alone for 24 h. **(C)** The cells in the AC groups were first pretreated with a gradient concentration of AC for 6 h, and then the cells in the CTX and AC groups were treated with CTX (300 μM) for 24 h. **(D)** The concentration of AMH in the supernatant of KGN cells. **(E)** Representative images of ROS levels as detected by DCFH staining in control, CTX and AC-treated KGN cells. **(F)** Fluorescence intensity of ROS signals. **(G)** Effects of AC on CTX-treated KGN cells mitochondrial membrane potential measured by JC-1 staining. Green fluorescence represents JC-1 monomers, whereas red fluorescence represents JC-1 aggregates. **(H)** Quantitative data of red/green fluorescence intensity ratio in the three groups *p < 0.05, ****p < 0.0001 vs. NC group. ##p < 0.01, ###p < 0.001 vs. CTX group.

The KGN cells were exposed to different levels of CTX or AC for 24 h, after which the cell viability was evaluated using the CCK-8 assay (Figures 5A–B). The findings indicated that the IC50 of CTX on KGN was approximately 500μM, and AC concentrations below 80 μM did not significantly affect KGN cell viability, suggesting that AC at concentrations below 80 μM is non-cytotoxic to KGN cells, and 300μM CTX was selected for subsequent experiments. Following this, KGN cells were treated with gradient concentrations of AC for 6 h and then induced with CTX (300 μM) for 24 h to establish DOR models (Figure 5C). The cell viability was assessed using the CCK-8 assay, which indicated a significant reduction in cell viability observed in the CTX group compared to the NC group, while the viability of KGN cells significantly improved upon treatment with different concentrations of AC. Notably, even at the lowest effective concentration of AC, 40μM, the AC group showed a significant recovery effect similar to that of the NC group. Therefore, a concentration of 40 μM AC was opted for further investigation. We examined the amount of AMH in the supernatants of the three groups of cells, NC, CTX, and AC, and the results showed that the secretion of AMH was significantly reduced in the CTX group compared with the NC group, while it was significantly increased in the 40 μM AC treatment group (Figure 5D). In addition, we examined the effects of AC on the ROS level and mitochondrial membrane potential (MMP) in CTX-treated KGN cells (Figures 5E–H). By DCFH staining, ROS levels were found to be raised in CTX-treated KGN cells. Treatment with AC for 24 h significantly reduced ROS level. Decreased mitochondrial membrane potential, which can be reflected by the decreased fluorescence intensity ratio of JC-1 monomers to aggregates, is an early indicator of apoptosis (Smiley et al., 1991). In normal KGN cells, the JC-1 probe mainly aggregated in the mitochondrial matrix in the form of aggregates and emitted red fluorescence with little green fluorescence. By contrast, treatment with CTX for 24 h significantly reduced red fluorescence and increased green fluorescence, suggesting the inhibited polymerization of JC-1 and mitochondrial membrane potential. Whereas, AC treatment moderated the mitochondrial depolarization induced by CTX. These proves the successful establishment of DOR cell model and the recovery of DOR granule cell function by AC.

### 3.5 Effect of AC on target proteins

**FIGURE 6:**
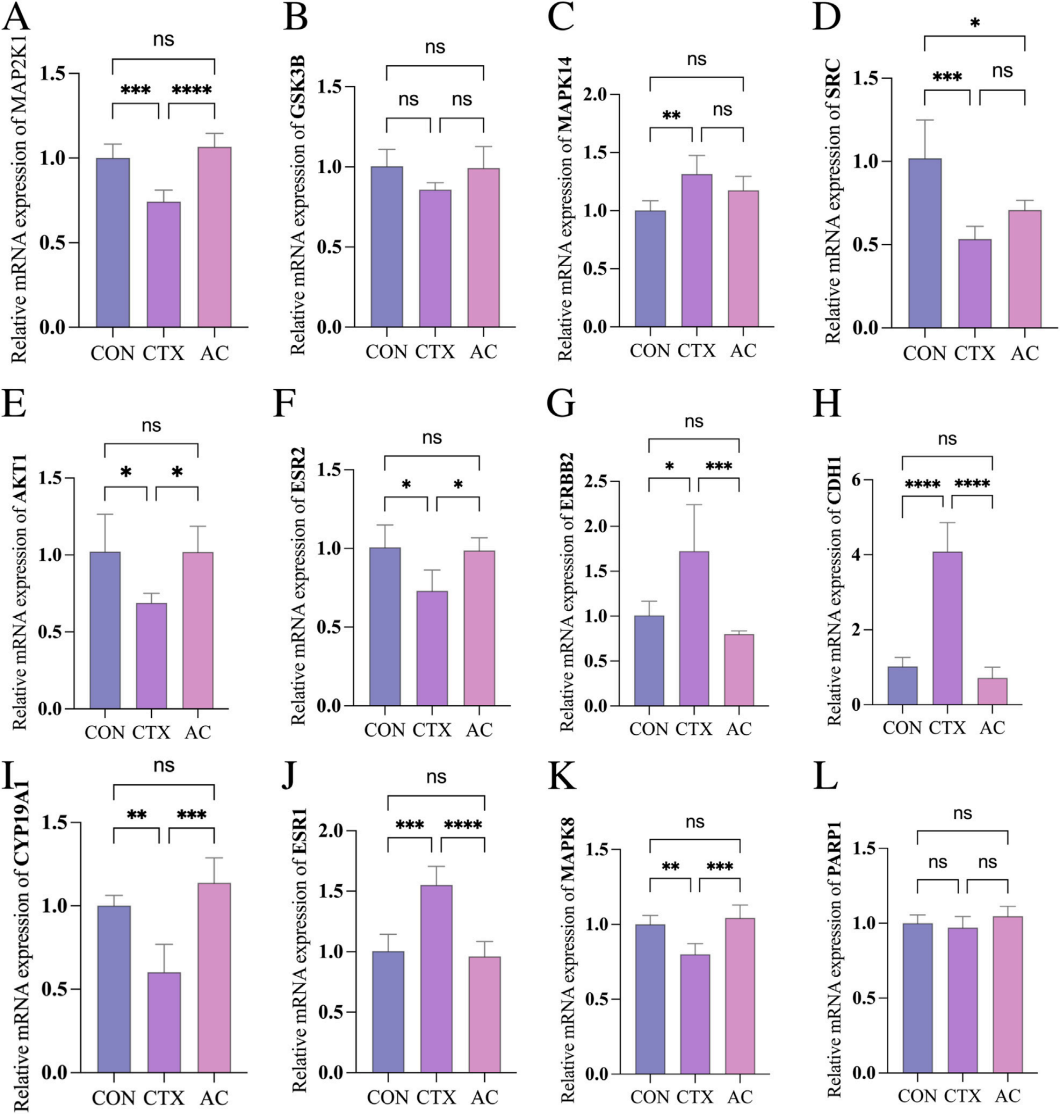
Effect of AC on target proteins. The mRNA expression levels of MAP2K1 **(A)**, GSK3B **(B)**, MAPK14 **(C)**, SRC **(D)**, AKT1 **(E)**, ESR2 **(F)**, ERBB2 **(G)**, CDH1 **(H)**, CYP19A1 **(I)**, ESR1 **(J)**, MAPK8 **(K)**, PARP1 **(L)**. AC treatment is able to reverse the abnormal gene expression of GSK3B, MAPK14, AKT1, ESR2, ERBB2, CYP19A1 and MAPK8 in the CTX group. NC: normal control, CTX: cyclophosphamide, AC: α-Cyperone, ns: P > 0.05, *P < 0.05, **P < 0.01, ***P < 0.001, ****P < 0.0001.

We have identified the top 12 genes with the strongest binding affinity based on our molecular docking findings. To assess the mRNA statement levels of the target proteins, we utilized RT-qPCR analysis in both the NC and CTX groups. The outcomes obtained from our experiments (Figure 6) demonstrated a significant increase in mRNA expression levels of MAPK14, ERBB2, CDH1, and ESR1 in the CTX group compared to the NC group. On the contrary, a significant reduction in the expression levels of MAP2K1, SRC, AKT1, ESR2, CYP19A1 and MAPK8 was observed in the CTX group compared to the NC group. AC treatment at 40 μM completely reversed these abreeant expression patterns for MAP2K1, AKT1, ESR2, ERBB2, CDH1, CYP19A1, ESR1, and MAPK8, restoring transcript levels close to—or even beyond—those of healthy controls. These directional changes imply that AC re-balances oestrogen synthesis and survival signalling within granulosa cells, providing a plausible transcriptional basis for its observed improvement in ovarian function.

### 3.6 LiP–MS analysis of identify AC-binding proteins in CTX-induced KGN cells

**FIGURE 7:**
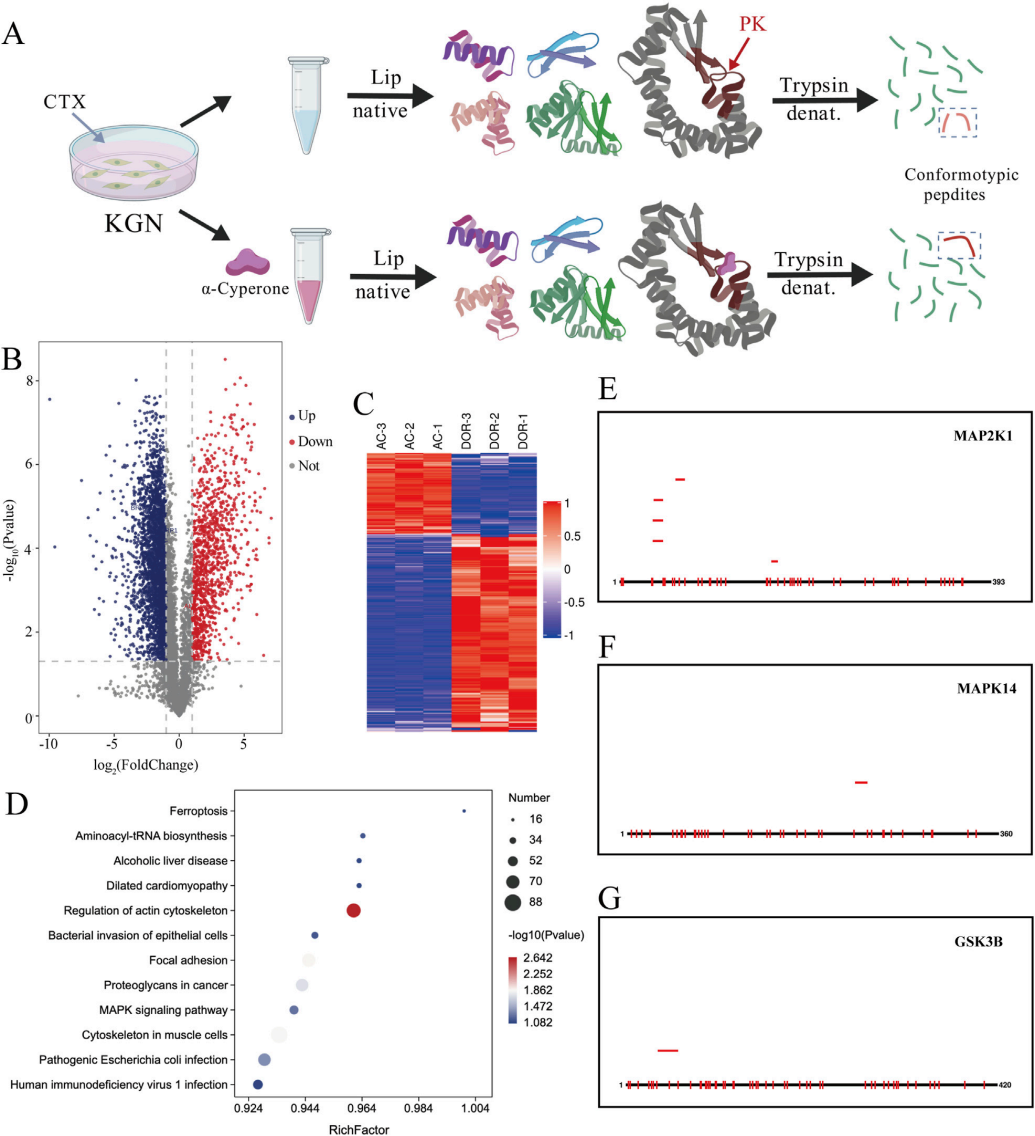
LiP–MS analysis of identify AC-binding proteins in CTX-induced KGN cells **(A)**. Schematic diagram of Lip-MS principle (Created with BioGDP.com). **(B)** The volcano plot of peptides in the two groups was plotted according to two factors: Fold change and P value (T-test). **(C)** Heatmap was used to identify differentially expressed peptides between the two groups. **(D)** KEGG analysis of differential peptides. **(E–G)** Distribution of differentially abundant peptides between DOR and AC groups. Black is the theoretical complete sequence; Red is the detected upregulated sequence. The sequence of the detected downregulated changes is shown in blue.

*In vitro*, limited proteolysis–mass spectrometry (LiP–MS) has provided a way to observe changes in both protein abundances and structures on a proteome-wide scale. We extracted whole-cell protein samples from CTX-induced KGN cells, divided the samples into two aliquots, and one sample was incubated with AC for 24 h. Two samples were treated with proteinase K (PK), and standard sample preparation was performed on two duplicate samples. All peptides were subjected to liquid chromatography-tandem MS (LC-MS/MS) in data independent acquisition (DIA) mode for DIA analysis (Figure 7A, Created with BioGDP.com (Jiang et al., 2025)).

In order to show the significant difference of peptides between the two groups, the volcano plot of peptides in the two groups was plotted according to two factors: Fold change and P value (T-test). The significantly downregulated peptides were marked in blue (FC < 0.6 7 and p < 0.05). Significantly upregulated peptides are marked in red (FC > 1.5 and p < 0.05) (Figure 7B). The asymmetric cut-offs were chosen to account for the higher technical variability observed in low-abundance peptides. Hierarchical Cluster was used to group and classify the differentially expressed peptides of the two groups, and the Heatmap was displayed. Based on the similarity basis, in the clustering grouping results, the data pattern similarity within the group is generally high, while the data pattern similarity between the groups is low, so the groups can be effectively distinguished. According to the screening criteria of fold change >1.5 times and P value < 0.05 (T-test or other), the significantly differentially expressed peptides could effectively separate the comparison groups (Figure 7C). In order to reveal the overall metabolic pathway enrichment characteristics of proteins corresponding to all differentially abundant peptides, proteins corresponding to all differentially abundant peptides were compared with all proteins of reference species (or all proteins identified experimentally) by KEGG annotation results (Figure 7D). The results showed that, the proteins corresponding to the differentially abundant peptides between the two groups were significantly enriched in Regulation of actin cytoskeleton, Focal adhesion, Proteoglycans in cancer, and Cytoskeleton in muscle cells and other pathways.

Based on the principle of limited enzyme digestion method to find small molecule binding targets, if the abundance of the long peptide of a sequence can be detected at the same time, and the abundance of the corresponding short peptide can be detected, or the short peptide on the corresponding sequence cannot be detected, it means that the sequence may be closed due to the binding of drugs, and the sequence is relatively likely to be the target sequence. By corresponding all detected peptides to the complete sequence of the protein, the situation of peptides with differential abundance on the protein and their position on the sequence can be visualized. We found that among the top three proteins with the highest binding energies in the molecular docking experiment (MAP2K1, MAPK14 and GSK3B, with PG. Cscore (50, 47 and 25, respectively) far above the background threshold (5), indicating the strongest intracellular affinity), significantly upregulated peptides could be detected, while no corresponding significantly downregulated peptides could be detected (Figures 7E–G). It can be understood that in the granule cells of the DOR group, these three proteins were digested into extremely short peptides so that they could not be detected, while in the cells of the AC-treated group, these three proteins could not be digested after binding to AC, so that the significantly upregulated long peptides could be detected. In other words, MAP2K1, MAPK14 and GSK3B may be the key targets of AC binding.

## 4 Discussion

The global decline in birth rates has intensified the infertility burden, with reproductive aging occurring at ever-younger ages. (Luo et al., 2021). Central to this trend is diminished ovarian reserve (DOR), a condition characterized by reduced oocyte quantity and quality that underlies ∼20% of ovarian disorders (Practice Committee of the American Society for Reproductive, 2012), 10% of general infertility and 31% of IVF cases. Without timely intervention, DOR can rapidly advance to premature ovarian failure (POF), underscoring the urgent need for effective therapeutic strategies. (Zhang et al., 2023; Lu et al., 2014; Christodoulaki et al., 2021).

Chronic inflammation and oxidative stress are now recognized drivers of DOR. Previous studies have demonstrated that administration of lipopolysaccharide (LPS) to mammals leads to hormonal dysregulation, reduced ovarian reserve, and infertility (Liebers et al., 2008). LPS, a widely used inflammatory trigger, suppresses estradiol synthesis, downregulates gonadotropin receptors and CYP19A1, and activates p38/NF-κB/JNK signaling in granulosa cells (GCs), reducing estradiol output and cell viability. These changes culminate in ovarian inflammation, fibrosis and GC apoptosis—changes that faithfully recapitulate the DOR phenotype in mice. (Shen et al., 2023; Magata et al., 2014; Sominsky et al., 2013; Shimizu et al., 2012; Herath et al., 2007). Because GCs constitute the largest steroidogenic population in the ovary and create the microenvironment required for follicle growth and oocyte maturation, their dysfunction directly compromises female fertility.

α-Cyperone, a major bioactive constituent of traditional Chinese medicines for gynecological disorders, has confirmed anti-inflammatory and antioxidant effects across multiple diseases (Taheri et al., 2021; Huang et al., 2023; Liu et al., 2019; Pei et al., 2020). Given the role of oxidative stress in the initiation and development of DOR, we postulate that α-Cyperone might exert a function in attenuating the decline of ovarian reserve and safeguarding ovarian function via its anti-inflammatory and antioxidant mechanisms. Consequently, we adopted the methodology of network pharmacology to predict the potential targets of α-Cyperone in the management of DOR. Yet our network-pharmacology analysis unexpectedly linked its potential efficacy in DOR to hormone-metabolism hubs rather than to classical oxidative-stress pathways. To dissect this paradigm, we substituted LPS with cyclophosphamide (CTX)—a DNA-alkylating agent that injures granulosa cells (GCs) without evoking overt inflammation—to exclude confounding antioxidant/anti-inflammatory effects. For this purpose, human KGN cells were pretreated with α-Cyperone (20 μM, 6 h) prior to CTX exposure, ensuring that any protective effect reflected preconditioning rather than post-injury rescue. Under this non-inflammatory DOR paradigm, α-Cyperone significantly restored AMH secretion, reduced ROS levels, stabilized mitochondrial membrane potential and improved cell viability. These data demonstrate that α-Cyperone preconditioning suffices to safeguard GC function against CTX-induced DOR injury, operating downstream—or in parallel to—oxidative stress and mitochondrial damage. The findings validate the hormone-centric prediction pipeline and position α-Cyperone as a metabolic modulator of ovarian reserve, warranting deeper dissection of its steroidogenic targets.

To further investigate the specific sites where α-Cyperone exerts its aforementioned functions, we conducted molecular docking validation. Generally, evaluating the outcomes of molecular docking primarily involves determining the minimum binding free energy (Delta G). Lower binding free energies (ΔG) indicate tighter, more stable complexes (Chen et al., 2023); accordingly, the top-ranked poses of AC against MAP2K1, MAPK14 and GSK3B exhibited robust negative ΔG values. These *in silico* predictions were corroborated by qPCR and LiP-MS, collectively implicating AC binding to these three kinases as a key mechanism for alleviating DOR.

MAP2K1 (Mitogen-Activated Protein Kinase Kinase 1) and MAPK14 (c-Jun N-terminal kinase) —core kinases of the stress-activated MAPK cascade—relay oxidative and inflammatory insults to the nucleus, driving granulosa-cell (GC) apoptosis and follicular depletion in DOR (He et al., 2020; Sun et al., 2021). Our docking and Lip-MS data show that α-cyperone binds directly to both proteins, restores their CTX-suppressed transcript levels (Figures 6, 7) and rapidly reduces ROS, providing a molecular explanation for the parallel recovery of AMH and mitochondrial membrane potential (Figure 5). Glycogen synthase kinase three beta (GSK3B), the third validated target, acts as a master switch that links Wnt, PI3K/AKT and NF-κB signaling to steroidogenesis and early embryogenesis (Liu et al., 2024; Zhang et al., 2022; Kang et al., 2025; Ahmadnia et al., 2024; Fu et al., 2023; Liao et al., 2025). Although AC occupies the catalytic pocket of GSK3B (Supplementary Figure S1) and reverses CTX-induced GC dysfunction, GSK3B mRNA levels remain unchanged (Figure 6), implying post-translational control: diminished kinase activity would stabilize β-catenin, enhance CYP19A1 transcription and suppress NF-κB-driven inflammation, all of which favor follicle survival (Ahmadnia et al., 2024; Liao et al., 2025).

In addition to the three targets confirmed by Lip-MS, seven others (MAPK8, AKT1, ESR1/ESR2, ERBB2, CDH1 and CYP19A1) responded to AC at the transcript level. We propose the following scenario, grounded on published signalling cascades. CTX-metabolite 4-HC inflicts DNA damage, represses mTOR and PI3K-AKT activity, their downregulation lowers AKT survival signalling and dampens the parallel JNK (MAPK8) and ERK (MAP2K1) cascades, collectively reducing the transcription of oestrogen receptor ESR2 and of the oestrogen-synthesising enzyme CYP19A1 (Mani et al., 2010; Baddela et al., 2025), leading to decreased granulosa-cell proliferation and oestradiol production—hallmarks of diminished ovarian reserve. Concurrently, cellular stress elicits a compensatory increase in growth-factor receptors and epithelial-mesenchymal transition genes, explaining the transient rise in ERBB2, CDH1 and ESR1 (Baddela et al., 2025; Xu et al., 2025). α-Cyperone has been reported to downregulate COX-2 and IL-6, to inhibit NF-κB signalling, and to re-activate the MAPK module, thereby restoring MAP2K1 expression and MAPK8 activity that drive granulosa-cell proliferation and differentiation (He et al., 2013; Luo et al., 2012; Jung et al., 2013). Concurrently, AC boosts the Akt/Nrf2/HO-1 axis, which rescues AKT1 expression, enhances antioxidant capacity and cell survival (Zhang et al., 2021; Chen et al., 2025), and indirectly increases ESR2 and CYP19A1 transcription, raising intra-follicular oestradiol (Mani et al., 2010). The relieved NF-κB drive allows ERBB2, CDH1 and ESR1 to return to baseline. This sequence integrates the qPCR changes into a coherent biological framework; detailed mechanistic validation is under way and will be reported separately.

Consequently, α-Cyperone may be a promising pharmacological agent that comprehensively regulates the aforementioned therapeutic targets in order to counteract the development of DOR. Derived from *Cyperus rotundus L.*—a common culinary spice that deodorises fish and meat while conferring anti-oxidant and anti-inflammatory benefits (Huang et al., 2023) —it is ideally positioned for early-stage dietary supplementation to delay ovarian reserve decline.

Although the parent herb Xiangfu (*Cyperi Rhizoma L*.) has long been used in gynaecology, the therapeutic value of α-cyperone in DOR has not been explored. Here, we integrate network pharmacology, molecular docking, *in vitro* assays and Lip-MS to identify and validate MAP2K1, MAPK14 and GSK3B as direct AC-binding targets in granulosa cells—an approach not previously reported. We further show that a 40 µM dose of AC restores AMH secretion, mitochondrial membrane potential and viability while suppressing ROS in a CTX-induced DOR model, revealing an antioxidant, pro-survival role for AC within the ovarian micro-environment. These findings extend the biological repertoire of AC to female reproductive biology and provide a new lead compound with defined targets for DOR intervention; mechanistic and translational studies now in progress will benchmark AC against existing therapeutics and refine its clinical dosing rationale.

However, the Lip-MS strategy used here is inherently constrained by detection thresholds. The method relies on the generation and ionisation of proteolytic peptides; consequently, low-abundance, transient, or weak-affinity interactors (Kd > 10 µM) are frequently missed, leading to false negatives. In addition, stochastic cleavage by proteinase K and variable peptide ionisation efficiency introduce background noise that can mask genuine AC-binding proteins. Rather than performing an unbiased intersection of all MS-detected bands with the 257 *in silico* candidates—which would dilute specificity with low-confidence peptides—we adopted a “filter-then-validate” pipeline: high-confidence targets derived from integrated network pharmacology (PubChem, PharmMapper, SwissTargetPrediction) plus molecular docking were subjected to Lip-MS, and only those yielding robust spectral counts (MAP2K1, GSK3B, MAPK14) were advanced for functional confirmation. This sequence minimises false positives while conserving experimental resources, and is widely employed in target-deconvolution studies to maintain both sensitivity and interpretability.

In addition, this study still has the following limitations. First, the study is explicitly hypothesis-generating, driven by network pharmacology: candidate targets were prioritized through *in silico* screening, molecular docking and Lip-MS, although network pharmacology, molecular docking and Lip-MS convergently nominated MAP2K1, MAPK14 and GSK3B as AC-binding targets, direct evidence that the compound activates or inhibits these kinases is still missing; Next, we are going to quantifying catalytic activity and phospho-status by SPR and Western blot, and we will initiate ChIP-qPCR to determine whether AC-induced kinase modulation alters transcription-factor occupancy on the promoters of MAPK8, AKT1, ESR2, CYP19A1, etc. Second, Although MAP2K1, MAPK14 and GSK3B appear able to bind α-cyperone and restore granulosa-cell function, definitive validation will require larger independent cohorts, gain-/loss-of-function models and quantitative proteomic analysis of patient follicular fluid. Third, α-Cyperone has not been benchmarked against established DOR therapeutics or tested in combination regimens; thus, its standalone or additive value beyond the observed antioxidant cytoprotection must be established through more comprehensive cellular, *in vivo* and clinical studies. Finally, the optimal dose, bioavailability and long-term cost-effectiveness of α-Cyperone remain undefined, and clinical translation may necessitate non-volatile delivery platforms such as engineered exosomes or nanoparticle systems. These issues are currently being actively studied and will be answered in subsequent research.

## 5 Conclusion

Our research utilizes a comprehensive methodology that includes network pharmacology, bioinformatics analysis, molecular docking simulation, *in vitro* cell assessments, and Lip-MS to extensively screen potential therapeutic targets for α-Cyperone against DOR. Furthermore, we demonstrate the protective effect of α-Cyperone on DOR granule cell function *in vitro*. The mechanism may be due to the targeted binding ability of AC to domains of MAP2K1, MAPK14 and GSK3B. These findings provide valuable insights into potential therapeutic targets for α-Cyperone against DOR and establish a solid theoretical foundation for its application in DOR treatment.

## Data Availability

The original contributions presented in the study are publicly available. Lip-MS raw data: [ProteomeXchange Consortium, dataset identifier PXD072555]. α-Cyperone chemical structure: PubChem CID 6452086 (https://pubchem.ncbi.nlm.nih.gov/compound/6452086). Protein sequences/structures: UniProt IDs listed in Table 2 (https://www.uniprot.org). Further inquiries can be directed to the corresponding author(s).

## Supplementary material

SUPPLEMENTARY FIGURE S1 Molecular docking validation of the binding capacity between AC and targets, and shows the surface structure of the target protein and the active pocket where AC is located. AC is shown in violet. The surface structure of the target protein is shown in gray. AC:α-Cyperone.

SUPPLEMENTARY FIGURE S2 Nodes in the DOR**(A)** and AC **(B)** PPI networks were colour-coded by source database (purple = PharmMapper, green = SwissTargetPrediction, blue = OMIM, pink = GeneCards).
