# Supplementary figures and images for: Identify the therapeutic role and potential mechanism of α-cyperone in diminished ovarian reserve based on network pharmacology, molecular docking, Lip-MS and experimental validation
Source: Front Pharmacol. 2026 Jan 15;16:1658536. doi: 10.3389/fphar.2025.1658536 (PMC12852401; doi:10.3389/fphar.2025.1658536)

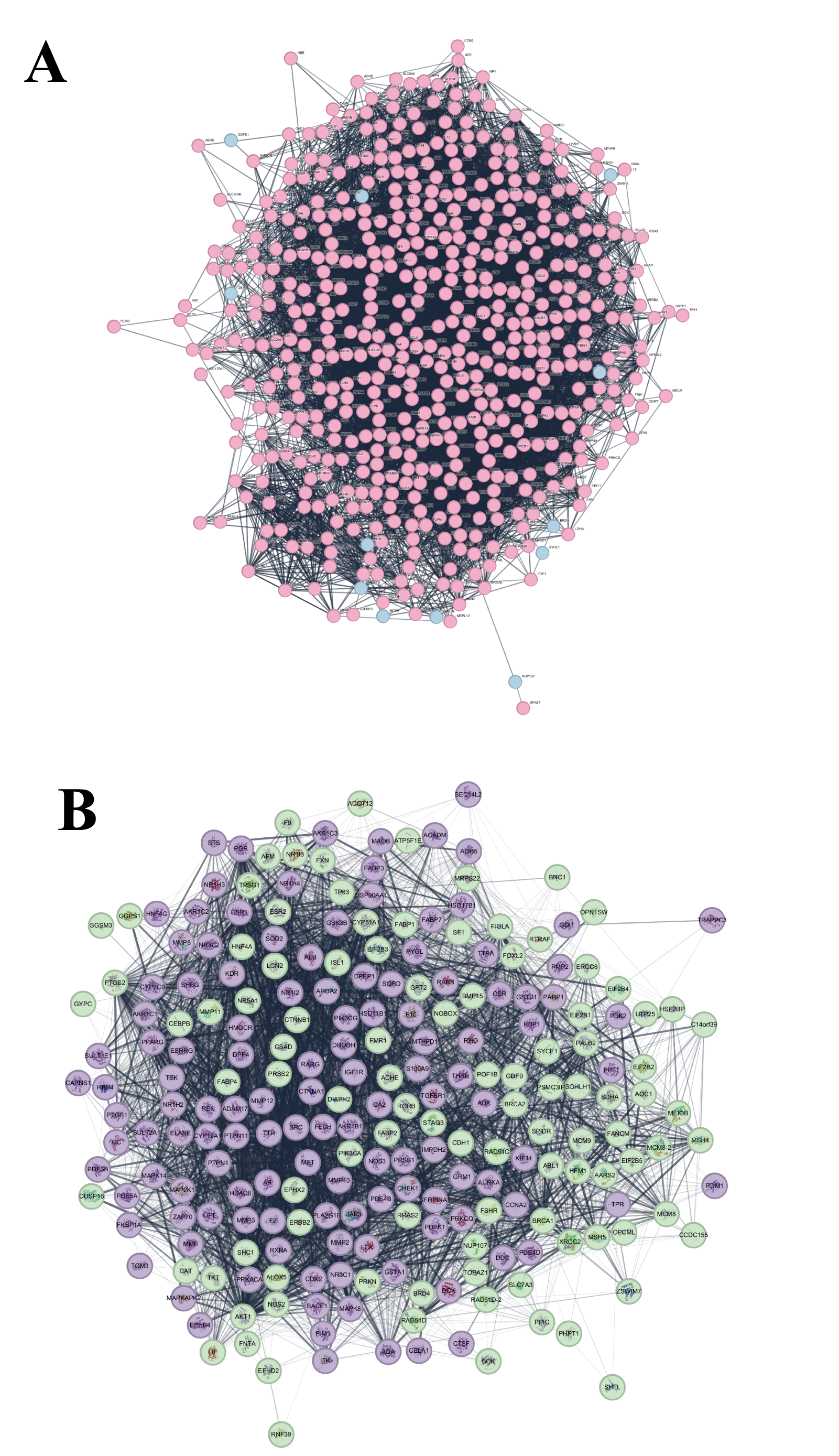

Supplement: Supplementary file 1 [file Image2.tif]

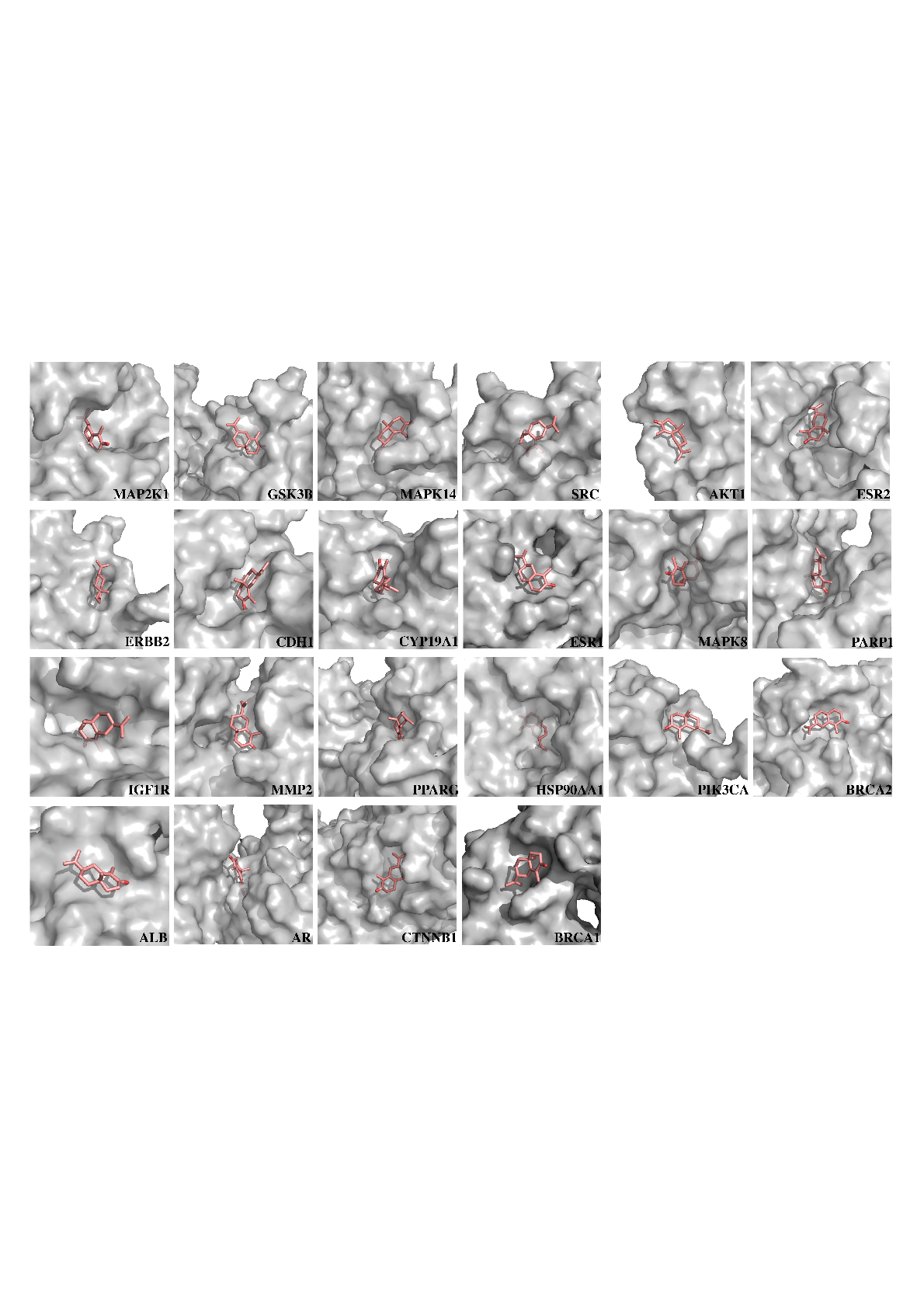

Supplement: Supplementary file 2 [file Image1.tif]
